# Cortical Network Overconnectivity Relates to Sensory, Cognitive, and Social Dimensions in Young Children With Autism Spectrum Disorder

**DOI:** 10.1002/aur.70315

**Published:** 2026-07-17

**Authors:** Borja Rodríguez‐Herreros, Ahmad Mheich, Joana Maria Almeida Osório, Sonia Richetin, Vincent Junod, Lorène Arnold, Victoria Aeschbach, Lilia Nora Adamou, Katherina Gschwend, Laura Mendes, David Romascano, Paola Yu, Marine Jequier Gygax, Anne M. Maillard, Micah M. Murray, Mahmoud Hassan, Nadia Chabane

**Affiliations:** ^1^ Service des Troubles du Spectre de l’Autisme et apparentés, Département de Psychiatrie Lausanne University Hospital (CHUV) Lausanne Switzerland; ^2^ Réseau Fribourgeois de santé Mentale (RFSM), Département de Psychologie Givisiez Switzerland; ^3^ Department of Radiology Lausanne University Hospital and University of Lausanne Lausanne Switzerland; ^4^ The Sense Innovation and Research Center, Lausanne and Sion Lausanne Switzerland; ^5^ MINDIG Rennes France; ^6^ School of Science and Engineering Reykjavik University Reykjavik Iceland

## Abstract

The heterogeneity in both the neurobiological mechanisms and the phenotypic presentations of autism spectrum disorder (ASD) poses a major challenge to clinical and translational research. Alterations in functional connectivity (FC) have been associated with ASD, yet it remains unclear whether and how divergent brain network properties may account for individual differences across ASD‐related symptomatology and behaviors. We applied source‐level reconstruction to rest‐like non‐task‐related high‐density EEG data in a cohort of 104 young children (38 with ASD) to identify global and local alterations of cortical network connectivity. We subsequently used regularized canonical correlation analysis (rCCA) to characterize specific FC patterns linked to variation in cognitive, social and sensory dimensions derived from standard clinical instruments. We found increased low‐frequency FC in frontotemporal cross‐hemispheric networks and lateral‐occipital regions of young ASD children versus healthy peers. RCCA revealed three distinct FC patterns in recurrent ASD‐related networks, each contributing to predict individual differences in cognitive, social and sensory features. These linked FC‐behavior dimensions may shed light on atypical brain network topology associated with specific phenotypic manifestations of ASD, which might implicate unique underlying neurobiological mechanisms.

## 1 Introduction

Autism spectrum disorder (ASD) is a neurodevelopmental condition classically characterized by persistent and significant social communication deficits and repetitive sensorimotor behaviors (American Psychiatric Association 2022). Understanding the early development of the neuronal circuitry underlying ASD is fundamental to elucidate its etiology and to promote more effective treatment interventions. Over recent years, the identification of pathological functional and structural brain networks has become one of the most promising prospects in the study of brain disorders (Fornito et al. 2015). Growing consensus suggests that disruptions in brain functional connectivity (FC) play an important role in the pathophysiology of ASD (Wang et al. 2013; Muller et al. 2011; Uddin et al. 2013). Several studies have reported that individuals with ASD exhibit FC alterations of large‐scale brain networks (Weng et al. 2010; Maximo et al. 2014; Holiga et al. 2019; Harikumar et al. 2021; Coburn et al. 2022). Yet, maturational changes in functional brain topology through early childhood and their association with clinically relevant ASD‐related phenotypic presentations remain poorly understood.

From a clinical perspective, there is considerable demand for non‐invasive, accessible methodological approaches that facilitate the earliest possible identification of brain functional alterations associated with ASD. High‐density resting‐state electroencephalography (HD‐EEG) has proven to be a practical and useful technique to explore large‐scale brain physiological activity at rest in pediatric clinical populations (Michel and Murray 2012; Ahmadi et al. 2021). Specifically, the convenience of resting‐state HD‐EEG to monitor brain activity in the absence of overt task performance or sensory stimulation stands out compared to other functional imaging methods that are rather difficult to implement with young ASD children. A considerable number of studies have investigated atypical brain FC in ASD individuals using rest‐like EEG protocols (Muller and Fishman 2018). Of the wide repertoire of EEG features, previous work has predominantly focused on the analysis of spectral power. These studies have reported inconsistent findings regarding altered brain connectivity in low (theta, alpha) and high frequency (gamma) band oscillations in ASD individuals (Dede et al. 2025). Reduced theta power has been associated with greater ASD symptomatology (Hornung et al. 2019), while local changes in alpha and gamma power have also been observed (Keehn et al. 2017; Maxwell et al. 2015). A relatively consistent profile of spectral power divergences in ASD children through development has emerged, suggesting excessive power in low and high frequency bands at early ages, but reduced power in the mid‐range band (Wang et al. 2013; Stroganova et al. 2007). Complementing these observations, a recent systematic review and meta‐analysis quantified reduced relative alpha and elevated gamma power in ASD, while noting substantial heterogeneity and sensitivity to paradigm and recording duration (Neo et al. 2023).

Besides spectral features, FC analysis has also been used to shed light on the brain network organization underpinning ASD. Several resting‐state EEG and magnetoencephalography (MEG) studies revealed characteristic altered network topology in children diagnosed with both syndromic and non‐syndromic ASD (Malaia et al. 2016; Peters et al. 2013; Tsiaras et al. 2011; Geng et al. 2023). There is a converging, though not uniform, trend suggesting reduced long‐range functional connectivity in ASD (O’Reilly et al. 2017). In contrast, evidence for increased short‐range connectivity remains inconsistent and insufficiently resolved. These studies support the developmental disconnection theory, which proposes a decreased long‐range integration accompanied by local overconnectivity and reduced functional specialization in the brain (Courchesne et al. 2007). Nevertheless, these EEG connectivity studies have mostly been performed in the sensor space (scalp level), thus using inflated connectivity estimates caused by volume conduction artifacts and preventing inferences about interacting brain regions (Nunez et al. 1997; Nolte et al. 2004). Moreover, efforts to associate clinical features with atypical brain network organization in ASD have been essentially addressed using resting‐state functional MRI (fMRI), with inconclusive evidence. For example, ASD core symptomatology and global cognition failed to be associated with several altered network metrics in ASD individuals older than 6 years (Keown et al. 2017; Sha et al. 2022). However, other studies reported that the balance of local and global efficiency between structural and functional networks was reduced in ASD adolescents, and inversely correlated with ASD symptom severity (Rudie and Dapretto 2013).

The present study seeks to determine how frequency‐specific connectivity patterns map onto distinct clinical features of autism. We conducted a source‐level assessment of early inefficiencies in brain network topology of young children with ASD. Furthermore, we assessed the clinical relevance of these network alterations by exploring generalizable linked dimensions with behavioral and clinical data. We measured FC using the “EEG source connectivity” method, which reduces the aforementioned volume conduction and allows networks to be identified directly at the cortical level with optimal spatiotemporal resolution (Hassan et al. 2014; Rizkallah et al. 2018; Mheich et al. 2020). We combined the use of HD‐EEG source connectivity methods with graph theory‐based analyses to characterize and quantify these functional networks at the cortical level. Finally, we used regularized canonical correlation analysis (rCCA) to assess the relationship among altered cortical network properties and cognitive, sensory, and social dimensions within the autism spectrum, to determine whether atypical brain network connectivity may account for individual differences across ASD‐related symptomatology and behaviors.

## 2 Materials and Methods

### 2.1 Participants

This study initially included 50 children diagnosed with autism spectrum disorder (ASD) and 73 typically developing (TD) children. However, HD‐EEG data from 12 children with ASD and seven TD did not pass the quality check and were excluded from analyses (see below for details), resulting in a final sample size of 104 participants (Table 1). All participants were aged from 2 to 8 years. All children in the ASD cohort were referred to the *Service des Troubles du Spectre de l'Autisme* at Lausanne University Hospital (STSA, CHUV). Trained licensed psychologists and child psychiatrists established formal ASD diagnosis based on the criteria of the Diagnostic and Statistical Manual of Mental Disorders, 5th edition, Text Revision (DSM‐5‐TR (American Psychiatric Association 2022)). The clinical procedure for the ASD diagnosis included a review of patients' medical and developmental history as well as the assessment with the Autism Diagnostic Interview–revised (ADI‐R (Rutter et al. 2003)), and the Autism Diagnosis Observation Scale‐2 (ADOS‐2 (Lord et al. 2012)). TD children were recruited from the general population through distribution of flyers to schools and pediatricians. Exclusion criteria for both groups included prematurity (< 36 weeks of gestation), a known neurological condition, neurodevelopmental disorder or learning disability, and specifically for the TD group a first‐degree relative diagnosed with ASD. The study was reviewed and approved by the *Commission Cantonale d'Ethique de la Recherche sur l'être humain* (CER‐VD, Switzerland), with the Project‐ID 2018‐00599. Written informed consent was obtained from participants' legal representatives before their participation.

**TABLE 1:** Demographic and clinical characteristics of the study cohort of TD and ASD children expressed as mean (SD).

		TD (*N* = 66)	ASD (*N* = 38)
Demographic	Age (y)	5.04 (2.1)	4.63 (2.2)
	Sex (F/M)**	30/36	6/32
ASD diagnosis	ADOS‐2 SA	—	12.1 (4.6)
	ADOS‐2 RRB	—	4.1 (1.8)
	ADOS‐2 total	—	16.3 (5.4)
Global cognition	Verbal IQ***	115.5 (14.1)	82.1 (24.4)
	Nonverbal IQ***	114.1 (13.5)	90.1 (19.1)
	Full Scale IQ***	115.3 (13.3)	82.7 (22.4)
Social cognition	SRS‐2 total raw score***	22.6 (12.4)	88.6 (26)
Sensory	SPM/SPM‐P total raw score***	71.3 (11.3)	103.5 (24.2)

Abbreviations: ADOS RRB, ADOS restricted and repetitive behaviors; ADOS‐2 SA, ADOS‐2 social affect; ASD, autism spectrum disorder; F/M, female/male; IQ, intelligence quotient; SPM, sensory processing measure; SRS‐2, social responsiveness scale‐2; y, years.

** *p* < 0.01.

*** *p* < 0.001.

### 2.2 Phenotypic Evaluation

Cognitive ability was measured using standardized tests according to the age and the developmental level of the participant; namely either the Wechsler Preschool and Primary Scale of Intelligence (WPPSI‐IV) (Wechsler 2013) or the Wechsler Intelligence Scale for Children, 5th edition (WISC‐V) (Wechsler 2014). Parents completed either the Preschool (2.5–4.5 years old) or the School‐age (4.5–18 years old) Social Responsiveness Scale 2nd edition (SRS‐2) (Constantino and Gruber 2012)—a 65‐item rating scale measuring deficits in social behavior associated with ASD—about their offspring. The Sensory Processing Measure (SPM) (Parham et al. 2007) and the Sensory Processing Measure—Preschool (SPM‐P) (Miller Kuhaneck et al. 2010) were the parent‐report questionnaires used to cover a range of behaviors and characteristics related to sensory processing, social participation, and praxis. Assessment, subscales, and standardized scores of the phenotypic evaluation are detailed in Supporting Information.

### 2.3 Electrophysiological Acquisition and Preprocessing

#### 2.3.1 Acquisition

HD‐EEG data were collected using a 128‐channel HydroCel geodesic sensor net together with Electrical Geodesics Inc. (EGI, Eugene, Oregon) Net Station software (Tucker 1993). HD‐EEG signals were sampled at 250 Hz. Passive, baseline, non‐task‐related HD‐EEG was collected during 5 min in a dark room as children watched an age‐appropriate soundless video presented on a computer screen while seated on a chair or on their caregiver's lap. In very young children, eyes‐open “rest” often yields excessive motion/eyeblinks and attrition. Naturalistic, low‐demand video viewing is an established workaround that reduces movement and improves compliance versus rest (Vanderwal et al. 2019). A low‐demand age‐appropriate video was used for each age range (2 to 8, by 1), and incorporated as a covariate in the analyses.

#### 2.3.2 Preprocessing

**FIGURE 1:**
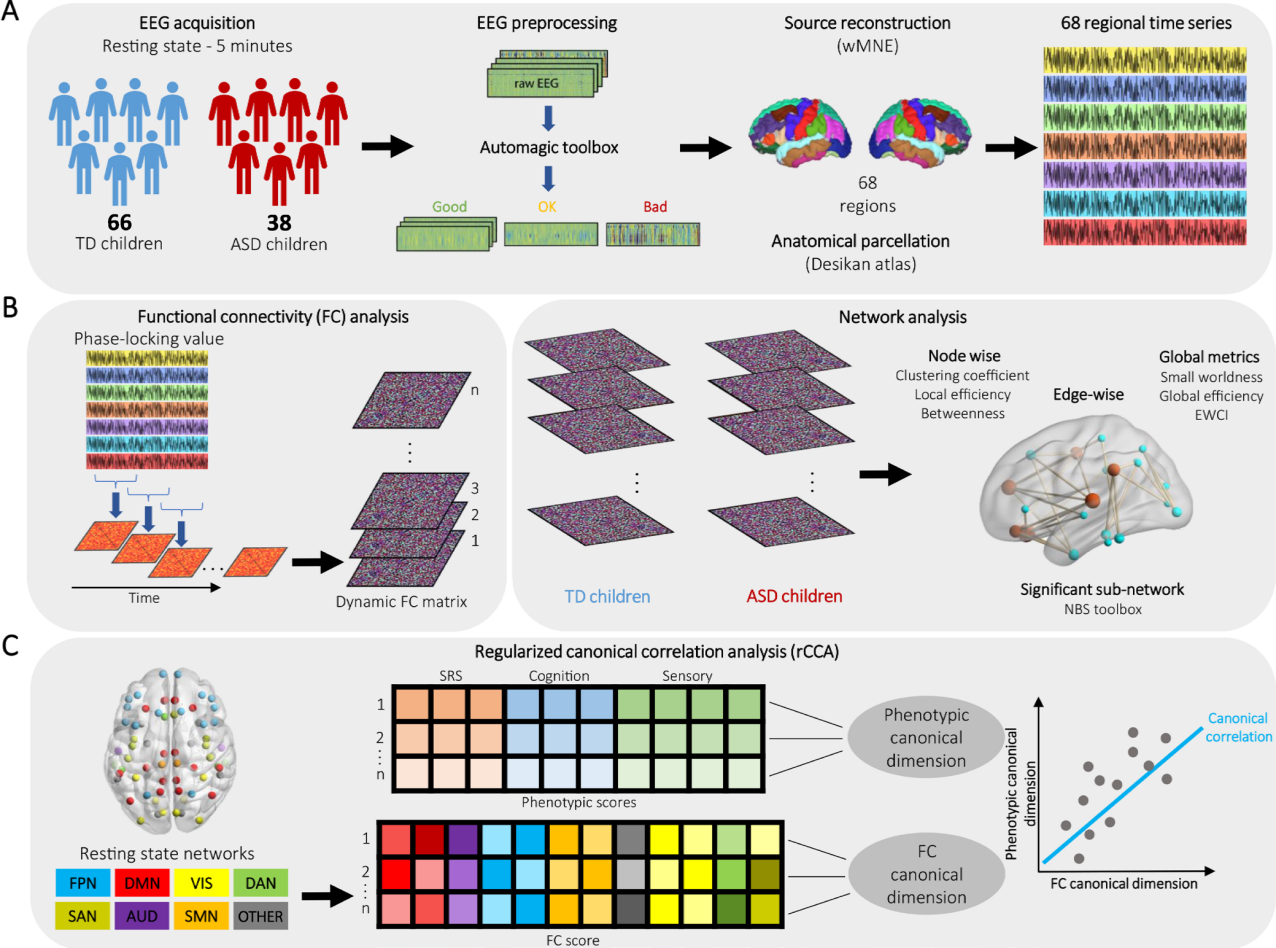
(A) Schematics summarizing pre‐processing pipeline and cortical parcellation. Automagic toolbox was used for quality assessment and artifact correction, and the cortical sources were reconstructed by solving the inverse problem. An anatomical parcellation was applied on the adapted MRI template yielding a time‐course RS HD‐EEG signal for 68 cortical regions of interest using the Desikan‐Killany atlas. (B) FC between the 68 regional time series was computed for each time window using the Phase Locking Value (PLV) method. A resulting dynamic FC matrix per individual was estimated by the standard deviation across all sliding windows. We compared dynamic FC matrices between ASD and TD young children using three levels of network analysis: (i) *node‐wise*: We computed metrics such as clustering coefficient (CC), local efficiency (LE), and betweenness centrality (BC); (ii) *edge‐wise*: We retrieved the cortical subnetwork whose edges underwent a significant change in FC values after statistical comparison of ASD and TD connectivity matrices using NBS approach; and (iii) *global network topology*: Small World Propensity (SWP), edge‐wise connectivity index (EWCI) and global efficiency (GE) were calculated to reflect overall network organization. (C) Regularized canonical correlation analysis (rCCA). The 68 parcellated cortical regions were grouped into eight large‐scale network communities as described in Figure S1. FC features for each of these network communities as well as cognitive, social and sensory phenotypic scores were entered into rCCA to search for a canonical pair that maximizes the correlation between FC and behavioral dimensions.

Raw HD‐EEG signals were bandpass filtered off‐line in the range 0.1–40 Hz. Filtered HD‐EEG data were further segmented into epochs of 40 s each, and the first segmented epoch was always discarded from later analyses. HD‐EEG epochs were preprocessed with MATLAB R2020b software (MathWorks) and the open‐source toolbox preprocessing pipeline Automagic (v2.5) (Pedroni et al. 2019). This toolbox simplifies and monitors artifact removal, interpolation of error‐prone channels and data quality rating (Figure 1A). The remaining artifact‐free epochs were ranked based on the overall high‐amplitude, and the first three epochs of 40 s were selected for analysis, in line with previous studies (Paban et al. 2019; Kabbara et al. 2017). A trained EEG expert (AM) reviewed the decisions made by Automagic, and the epoch selection process underwent confirmation through visual inspection. Over 84% of the HD‐EEG data exhibited “ok” or “good” data quality. As a result, a total of 12 children with ASD and seven TD children exhibited HD‐EEG signals categorized as “bad” and thus met criteria for exclusion of later analyses.

#### 2.3.3 Functional Connectivity (FC)

Details on source reconstruction are provided in the Supporting Information. The second step of the EEG source connectivity method involved the estimation of the statistical couplings between the 68 source‐reconstructed regional time series separately for four different frequency bands: theta (4–8 Hz), alpha (8–13 Hz), beta (13–30 Hz), and gamma (30–45 Hz). FC between the regional time series was computed for each frequency band using the Phase locking value (PLV) method (Lachaux et al. 1999). This measure, ranging between 0 and 1, reflects interactions between two oscillatory signals through quantification of the phase relationships. The combination of the wMNE and the PLV methods was chosen according to a recent model‐based comparative study of different inverse/connectivity combinations (Hassan, Merlet, et al. 2017). We obtained for each participant four dynamic FC matrices in each frequency band, ultimately averaged across time and epochs to obtain a single static FC matrix per frequency band. This resulted in a 68 × 68 symmetrical FC matrix from which 68 × 67/2 = 2278 unique connections were obtained in each frequency band, representing the pairwise connections between all of the cortical ROIs (Figure 1B).

#### 2.3.4 Network Metrics

FC matrices were characterized using network measures derived from graph theory, in a system in which nodes (68 cortical regions) are connected by edges reflecting the functional and/or effective connections. Then, network topological properties were quantified to characterize brain network architecture using the Brain Connectivity Toolbox (BCT) (Rubinov and Sporns 2010) and visualized with BrainNet viewer (Xia et al. 2013). Functional networks were analyzed at three different levels: brain regions (node‐wise), connections (edge‐wise), and global (the whole brain). The equations to compute all network metrics described below are detailed in Supporting Information.

##### 2.3.4.1 Node‐Wise

We measured clustering coefficient (CC), betweenness centrality (BC), and local efficiency (LE). CC is a major metric for determining the level of information segregation of the network by showing how many nodes are clustered together in a graph (Rubinov and Sporns 2010). BC of a node indicates the importance and the amount of information flowing via that node (Boccaletti et al. 2006). LE is an average measure of efficiency of information transfer limited to neighboring nodes, defined as the average inverse SPL of all neighbors of a given node among themselves (Cohen and D'Esposito 2016).

##### 2.3.4.2 Edge‐Wise

Network metrics were characterized using the Network‐Based Statistic (NBS) toolbox (Zalesky et al. 2010). To do that, an ANCOVA analysis was fitted to each of the 2278 phase synchronization values of each of the edges, yielding a *p*‐value indicating the probability of rejecting the null hypothesis at each edge. Selecting the NBS threshold represents an arbitrary parameter, although control of family‐wise error rate is guaranteed irrespective of the threshold choice. A range of component‐forming thresholds was then applied to the test statistic (*t*‐values; 1.0–4.5, step 0.1) to define suprathreshold connections. The size of the components was then compared to a null distribution of maximal component sizes obtained using 5000 permutations testing to obtain *p*‐values corrected for multiple comparisons (Zalesky et al. 2010). We chose a compromise threshold to select the significant NBS network (*p* < 0.05), involving a number of nodes closest to 75% (51/68) of total network nodes, in line with previous studies (Conti et al. 2023).

##### 2.3.4.3 Global Topology

Small World Propensity (SWP) (Muldoon et al. 2016) has been proposed as a reliable measure to estimate information transfer in a network. SWP evaluates the level of small‐world structure between any two nodes, that is, higher local clustering than a random network, yet short average path length. Furthermore, we measured global efficiency (GE) to further evaluate differences in the integration of the neural networks of children with ASD and TD children. GE measures the efficiency of information transfer among all pairs of nodes in the graph and thus reflects the efficiency of interaction across the whole graph (Rubinov and Sporns 2010; de Pasquale et al. 2016). When applying NBS to compare the networks of the two groups, we computed an Edge‐wise Connectivity Index (EWCI), defined as the sum of the weights of the resultant subnetwork (Hassan, Chaton, et al. 2017) (see Supporting Information).

#### 2.3.5 Statistical Analysis

Shapiro–Wilk normality tests and Levene's tests were conducted to assess the normality and homogeneity of variance prior to group comparisons. When normality was not met, Wilcoxon signed‐rank tests were used. Group differences of power spectral density and network metrics between ASD and TD children were assessed using linear models with covariates in R (v4.2.1) (R Core Team 2013). We performed covariate adjustment prior to group comparisons. Specifically, a linear regression model was fitted with each dependent variable and age, sex, and FSIQ as predictors, estimated across all participants regardless of group. Adjusted values were then derived as the residuals from this model, re‐centered by adding the grand mean of the raw scores, so that the adjusted values remained on the original scale. Group differences in adjusted values between ASD and TD children were subsequently assessed using a linear model including Group, age, sex, and FSIQ as predictors, with the Group coefficient providing the estimate of the between‐group difference after covariate control. To assess the developmental trajectory of global network metrics as a function of age in the TD and ASD groups, we generated partially adjusted values of network metrics (GE, SWP, EWCI) by regressing out sex and FSIQ only, deliberately retaining age so as not to artificially suppress the age‐related signal of interest. The resulting values were re‐centered to the grand mean and used as the dependent variable in the developmental curve analysis. Developmental trajectories were visualized separately for each group using locally estimated scatterplot smoothing (LOESS), providing a flexible, non‐parametric representation of the age‐related trend without assuming linearity. Spearman rank correlations were computed separately for each group to assess the monotonic relationship between age and adjusted scores, with *p*‐values estimated using a normal approximation to account for tied values. Finally, we performed a quantitative node‐wise analysis on the brain networks of TD and ASD children using BC, CC, and LE as network metrics. *p*‐values for node features were corrected for multiple comparisons using FDR across the 68 nodes for each metric. To further explore how edge‐wise differences might manifest in pairwise—ROI to ROI—connectivity strengths, we tested for FC differences in each of the 2′278 possible connections. *p*‐values were corrected by permutation test using NBS. Effect sizes are reported as well in the form of Cohen's *D* for two‐sided Welch's *t*‐tests, and standardized beta values for regressions. We generated Cohen's *d* maps from FDR‐corrected *t* scores to show the unbiased magnitude of the effects. All statistical analyses were conducted using methods robust to unequal group sizes.

### 2.4 Regularized Canonical Correlation Analysis (rCCA)

Canonical correlation analysis (CCA) is a multivariate technique for identifying linear relationships between two sets of variables. To mitigate instability of weight estimates and inflated out‐of‐sample correlations that can arise with low sample‐to‐feature ratios (Helmer et al. 2024), we employed regularized CCA (rCCA). rCCA imposes an L2 penalty (λ) on the canonical vectors and shrinks the within‐set covariance estimates, thereby reducing variance relative to the unregularized solution (Vinod 1976; Leurgans et al. 1993). We used rCCA to identify latent generalizable brain–behavior linked dimensions explaining individual differences in three ASD‐related domains: social, cognitive, and sensory processing behaviors (Figure 1C). We performed three L2‐regularized CCA using the rCCA function in R mixOmics package (Rohart et al. 2017), separately for the three abovementioned clinical domains.

We implemented a robust selection of clinically meaningful FC features and define a low‐dimensional representation to avoid potential overfitting and handle multicollinearity in the input variable sets, as detailed in Supporting Information. To determine the cortical networks that are highly contributing to the identified FC canonical dimensions, the ranked FC loadings were parcellated and assigned to one of the eight well‐established large‐scale cortical within‐ and between‐network communities (Figure S1; Table S1) (Mohr et al. 2016): visual network (VIS), auditory network (AUD), sensorimotor network (SMN), fronto‐parietal network (FPN), salience network (SAN), dorsal attentional network (DAN), default mode network (DMN) and other networks (OTHER). FC loadings assigned to each network community were summed as measures of network strength (Pan et al. 2023, 2024), establishing the final number of FC features incorporated to the rCCA in (8 × 8/2 = 32). We generated two canonical variate pairs (FC and behavior scores) with values for all participants and a canonical correlation for each variate pair. To illustrate the features that contribute to each canonical dimension, we computed regression coefficients (i.e., canonical loadings) between each canonical dimension and its original variables (Drysdale et al. 2017).

## 3 Results

### 3.1 Demographics and Clinical Characteristics

Demographics and clinical scores are presented in Table 1. Age was not significantly different between TD and ASD children (*p* = 0.35), but we observed a higher prevalence of males in the ASD group (*χ* ^2^ = 8.1, *p* = 0.005). ASD children had a significantly lower global cognitive functioning compared to TDs, with an average of 33 points lower VIQ (*p* = 6.3e−10) and a 24‐point lower NVIQ (*p* = 8.3e−9). ASD children scored higher in both the SRS‐2 (*p* = 1.02e−17) and the SPM total score (*p* = 1.26e−9). SRS‐2 and SPM subscales scores are detailed in Table S2.

### 3.2 Frequency‐Based Analysis

**FIGURE 2:**
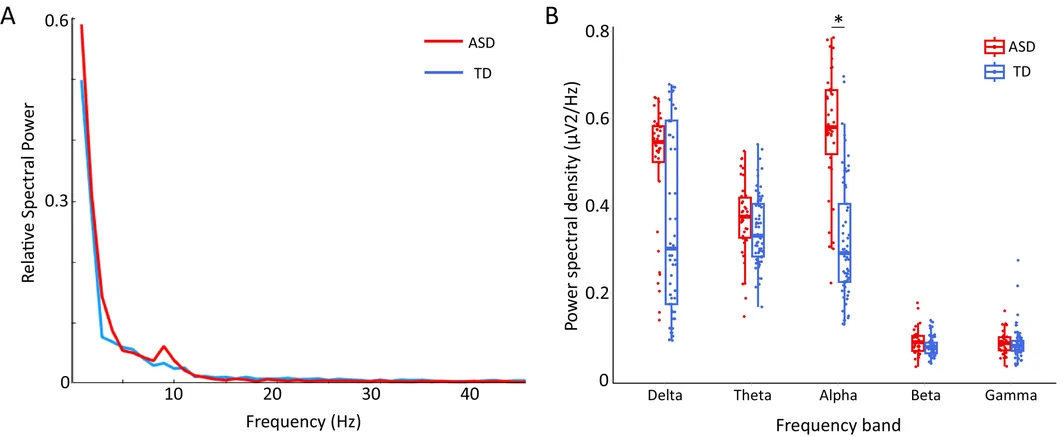
(A) Power spectral representation of EEG data for ASD (red) and TD (blue) young children. (B) Frequency‐based analysis: Individual values of the power spectral density for ASD and TD young children at five frequency bands: [delta (0.5–4 Hz); theta (4–8 Hz); alpha (8–13 Hz); beta (13–30 Hz); gamma (30–45 Hz)]. In all boxplots, the thick horizontal line shows the median, and the bottom and top of the box show the 25th (quartile 1 [Q1]) and the 75th (quartile 3 [Q3]) percentile, respectively. The upper whisker ends at highest observed data value within the span from Q3 to Q3 + 1.5 times the interquartile range (Q3–Q1), and lower whisker ends at lowest observed data value within the span for Q1 to Q1−(1.5 * interquartile range). Points not reached by the whiskers are outliers. Significant post hoc group comparisons are represented by solid lines above. *p < 0.01, Bonferroni‐corrected.

To validate the consistency of our EEG data spectral power, we analyzed the overall changes in the relative spectral power of ASD and TD children over all brain regions (Figure 2A). We confirmed that the peak power fell within the alpha band for both groups, as expected in task‐free EEG recordings (Grandy et al. 2013). Higher relative power seems to characterize the neural oscillatory activity of ASD children at lower frequency bands. Figure 2B summarizes the frequency‐based analysis, showing an increase in the power spectral density of ASD children in the delta and alpha frequency bands, although only the differences in the alpha band survived correction for multiple comparisons (*p* = 1.5e−12, Bonferroni corrected). We found no significant differences in theta, beta, or gamma frequency bands. The results presented in the following sections are then circumscribed to the alpha band (8–13 Hz).

### 3.3 Mapping of Cortical Topological Differences

#### 3.3.1 Node‐Wise

**FIGURE 3:**
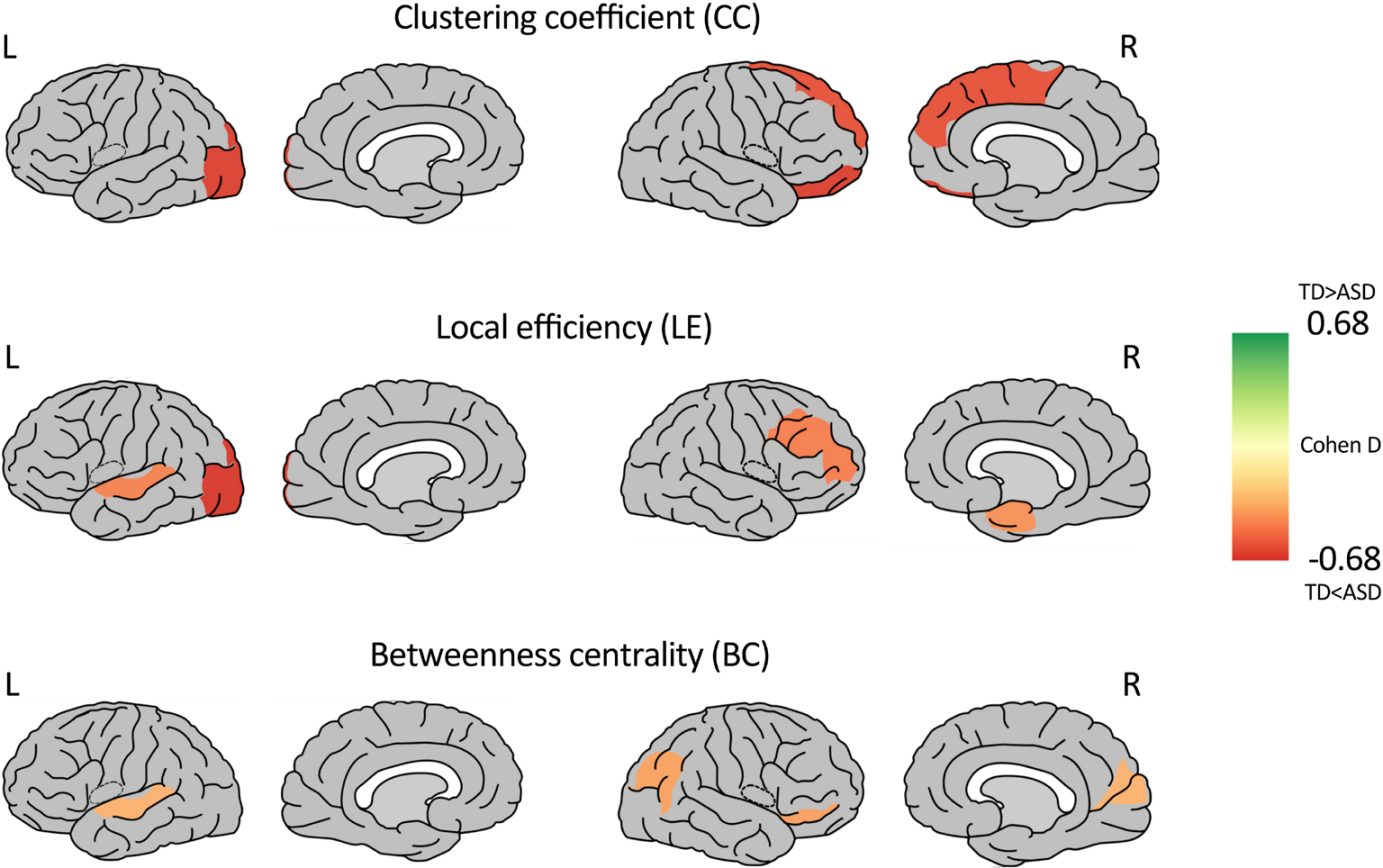
Node‐wise analysis. Cortical mapping of local network overconnectivity in young children with ASD. Cohen's *d* maps from FDR‐corrected *t* scores in clustering coefficient (upper), local efficiency (middle), and betweenness centrality (lower) are displayed. Color bars represent Cohen's *d*. L, left; R, right.

Differences in node‐wise network metrics between ASD and TD children are displayed in Figure 3, after controlling for age, sex, and FSIQ. The results highlight higher CC in the network nodes corresponding to frontal areas—the right lateral and superior orbitofrontal cortex (*d* = −0.34, *p* = 0.03)—as well as the left lateral occipital cortex (*d* = −0.52, *p* = 1e−3) of ASD children. These results present a distinct symmetry between the two hemispheres. We also found that ASD children exhibited higher CC in the precuneus, a part of the superior parietal lobule, of the right hemisphere (*d* = −0.25, *p* = 0.03), but this result lost statistical significance when FSIQ was added as a covariate. Similarly, we found the largest differences in LE also in the left lateral occipital region (*d* = −0.52, *p* = 1.2e−3). Lastly, we found significantly higher BC in both the right inferior parietal and the rostral part of the inferior frontal gyrus of ASD children (*d* = −0.27, *p* = 0.01). Thus, network nodes with significantly different BC do not seem to be symmetrically distributed across the hemispheres, but involve predominantly the right one. Smaller trends in the left parsopercularis and superior temporal gyrus did not persist after FDR‐correction (Table 2).

**TABLE 2:** Node‐wise results: List of cortical regions with significantly different network metrics between TD and ASD children expressed as mean (SD) adjusted for age, sex and FSIQ.

		TD	ASD	Cohen *D*	*p*
CC	R lateral orbitofrontal	0.62 (0.03)	0.66 (0.06)	−0.51	0.02
	R superior frontal	0.63 (0.03)	0.66 (0.07)	−0.47	0.04
	L lateral occipital	0.59 (0.04)	0.64 (0.06)	−0.52	0.01
LE	R rostral middlefrontal	0.63 (0.03)	0.67 (0.06)	−0.46	0.02
	R entorhinal	0.59 (0.03)	0.63 (0.07)	−0.52	0.01
	L superior temporal	0.63 (0.04)	0.66 (0.06)	−0.43	0.04
	L lateral occipital	0.61 (0.03)	0.64 (0.07)	−0.36	0.04
BC	R inferior parietal	0.0064 (0.01)	0.0098 (0.01)	−0.27	0.01
	R cuneus	0.004 (0.01)	0.0066 (0.01)	−0.22	0.03
	R pars orbitalis	0.003 (0.01)	0.0018 (0.02)	−0.28	0.03
	L superior temporal	0.009 (0.01)	0.0071 (0.02)	−0.22	0.03

#### 3.3.2 Edge‐Wise

**FIGURE 4:**
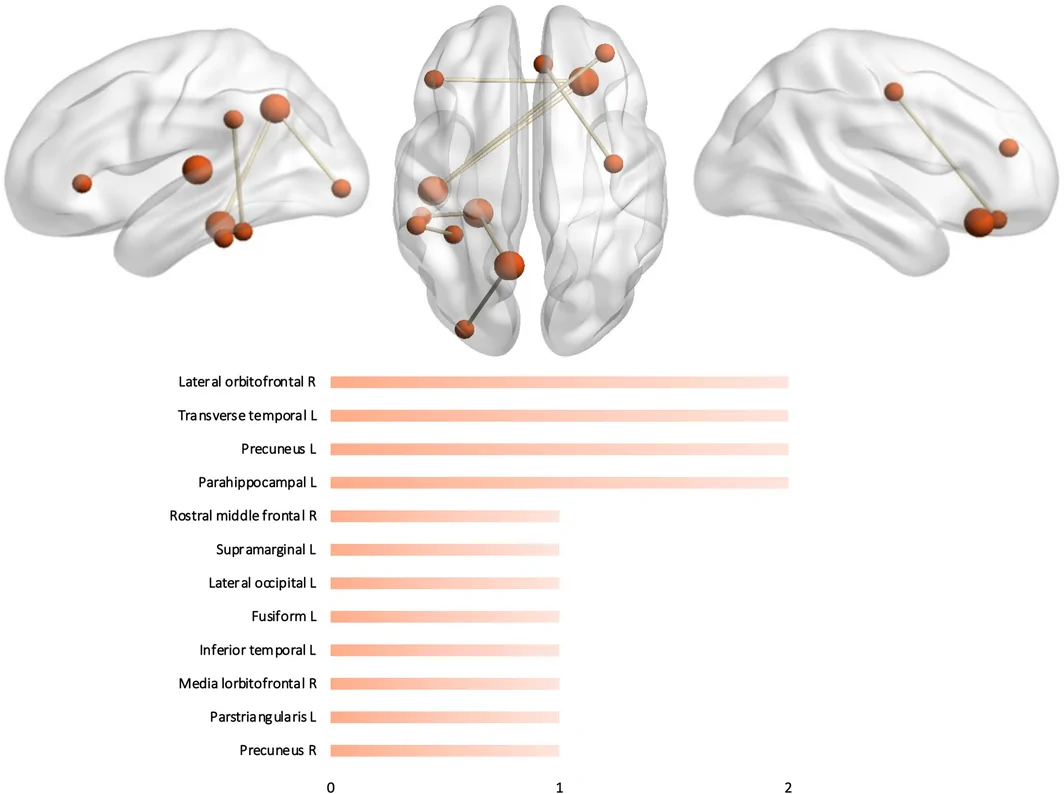
Edge‐wise analysis. Graphical representation (upper)of the resulting network showing the 30 FDR‐corrected edges with higher FC in young children with ASD. Each cortical region (node) is represented as a sphere plotted according to the stereotactic coordinates of its centroid, and each suprathreshold edge represents the connections between the regions as a solid line. The size of the node is proportional to its degree, that is, the number of connections in which it takes part. In the lower panel, representation of the degree for each brain region identified in the resulting network. *p*‐values were corrected by permutation test using NBS (*p* < 0.05). L, left; R, right.

The results of the edge‐wise analysis after adjustment for age, sex, and FSIQ are summarized in Figure 4. The network reconfiguration in the alpha band that emerged when comparing ASD and TD children revealed a single connected subnetwork of significantly hyperconnected functional connections in ASD children, comprising 12 nodes and 11 edges (*p* < 0.05 after correction for multiple comparisons). The implicated regions spanned frontal, temporal, parietal, and occipital cortices, with a predominantly left‐lateralized distribution. The resulting subnetwork was anchored by several hub‐like nodes with multiple connections, most notably the left parahippocampal gyrus, left precuneus, and left transverse temporal gyrus. The right lateral orbitofrontal cortex also emerged as a prominent node, presenting connections across the hemispheres, as well as intra‐hemispheric connections on the right anterior and left posterior sides. To better characterize the mapping of the edges, we calculate the degree for each of the 12 brain regions within the resulting network, which represents the number of connections (edges) associated with each specific node. We confirmed that the abovementioned regions have the highest connection density (degree = 2) in the resulting subnetwork with significantly increased FC in ASD children.

### 3.4 Group Differences and Developmental Trajectories of Global Network Metrics

**FIGURE 5:**
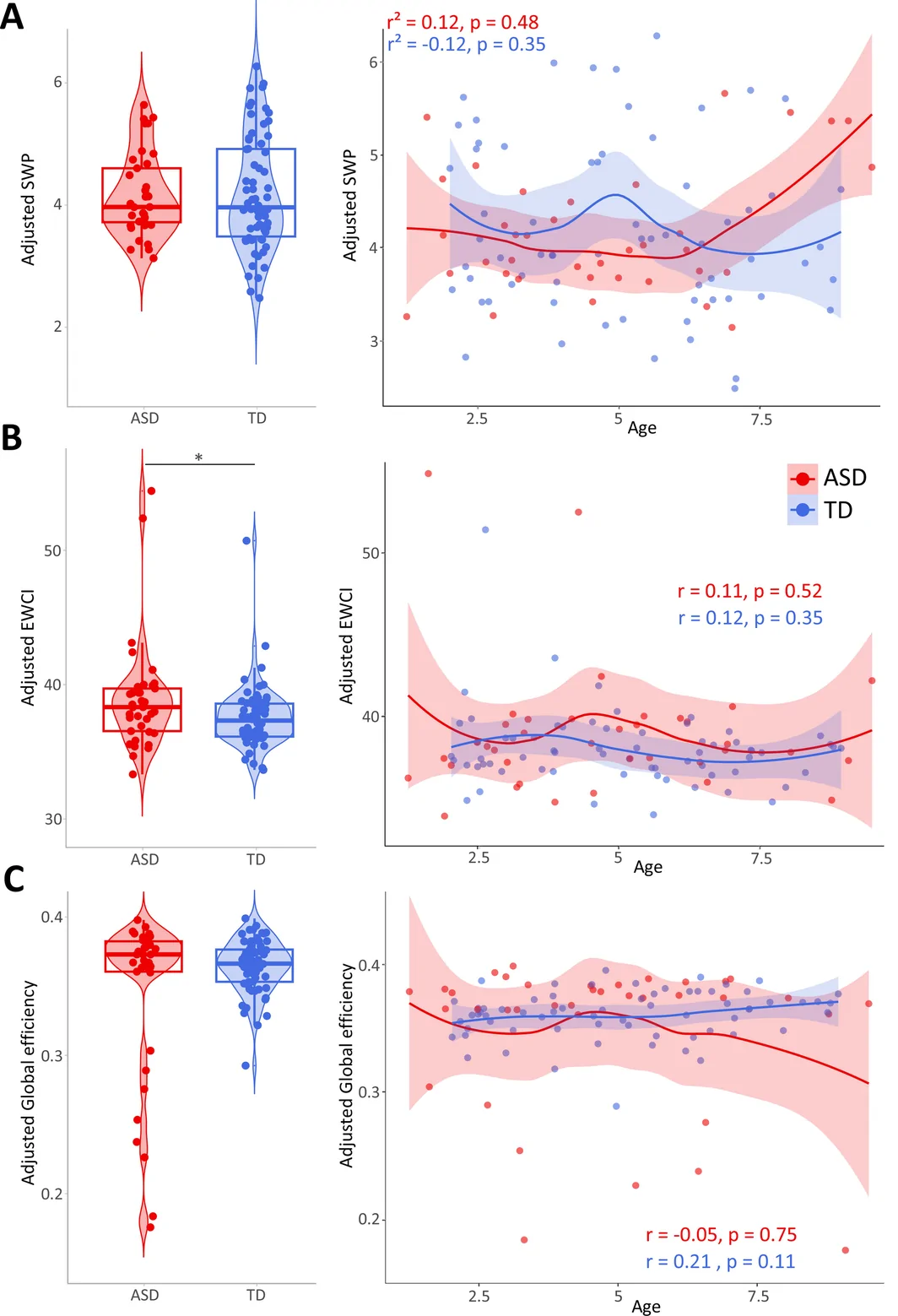
Global network topology. For each metric (A, small‐world propensity; B, EWCI; C, global efficiency) the left panel shows group comparison of individual values between ASD and TD young children, whereas the right panel displays age‐related changes in global network topology for both groups. EWCI was computed from the resulting network of the edge‐wise analysis. Linear and quadratic (2nd order) regressions of age were modeled for each global network metric. In all boxplots, the thick horizontal line shows the median, and the bottom and top of the box show the 25th (quartile 1 [Q1]) and the 75th (quartile 3 [Q3]) percentile, respectively. The upper whisker ends at highest observed data value within the span from Q3 to Q3 + 1.5 times the interquartile range (Q3–Q1), and lower whisker ends at lowest observed data value within the span for Q1 to Q1−(1.5 * interquartile range). Points not reached by the whiskers are outliers. **p < 0.01, FDR‐corrected.

Global estimates and developmental trajectories of the analyzed network metrics are presented in Figure 5. The group comparison showed that SWP was comparable between ASD and TD children (*t*_(103)_ = 0.25, *p* = 0.8, Figure 5A). We computed the EWCI using the resulting network from the edge‐wise analysis consisting of 11 edges and 12 brain regions. EWCI was significantly higher in ASD compared to TD children, even after controlling for FSIQ differences (*t*_(103)_ = 2.3, *p* = 0.02, Figure 5B). Lastly, GE presented a trend to be higher in ASD children (*t*_(103)_ = 2.17, *p* = 0.033), but did not survive FDR correction (Figure 5C). The effects remained unchanged when outliers (> 2.5 SD) were excluded. The analysis of the developmental trajectory of these network metrics revealed that the increased EWCI was steadily constant and already present at an early age. We also observed that SWP did not significantly change with age in TD children, but pointed to an increase in SWP at older ages. Finally, GE showed no change over time in both ASD and TD groups.

### 3.5 Linked Brain‐Behavior Dimensions

**FIGURE 6:**
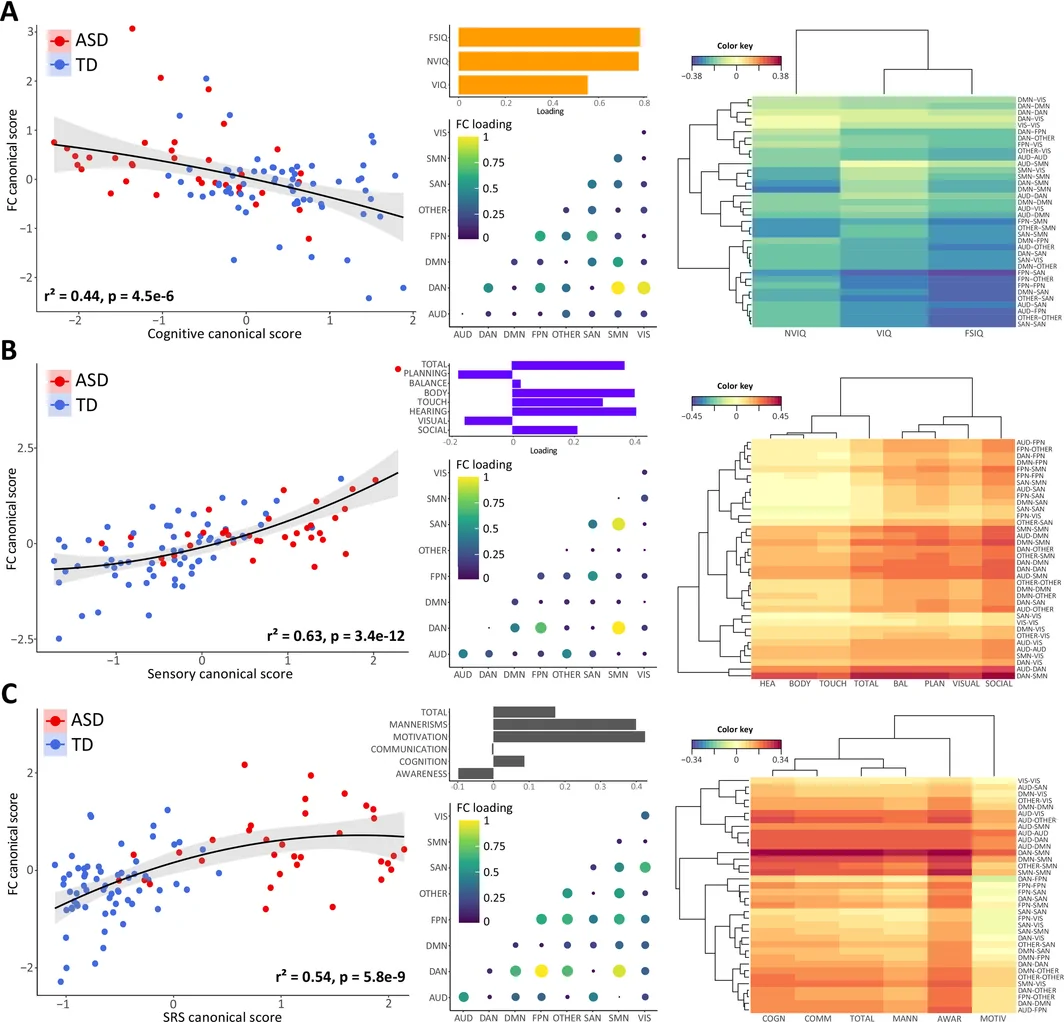
Linked brain–behavior dimensions to predict individual differences in cognitive (A), social (B), and sensory processing (C) scores using network connectivity patterns in young ASD and TD children. Left panels are scatterplots depicting linear correlations of FC with behavioral scores: Cognitive (*r* ^2^ = 0.44), social (*r* ^2^ = 0.63), and sensory processing (*r* ^2^ = 0.54). The middle panel shows the loadings of behavioral and FC features, separately for each brain and behavior dimension. On the right, Clustered Image Maps representing Pearson correlation coefficients between FC and behavior datasets. Hierarchical clustering is applied on the FC (rows) and behavior (columns) features simultaneously. Dendrograms are used along the axes to depict how each row/column clusters based on the hierarchical clustering method.

We conducted three rCCA as a data‐driven approach to unfold linked dimensions between brain FC and social, cognitive and sensory processing features, respectively, in both ASD and TD children. Following the grid search of optimized regularization parameters of rCCA (Figure S2), we selected for further analysis the canonical pairs based on the accumulated covariance explained. For the FC‐cognition rCCA, the most significant canonical variate pairs (*r* ^2^ = 0.44, p_FDR_ = 4.5e−6) explained 67% of variance of the cognitive features, and 17% of variance of FC features, respectively (Figure 6A). We observed that children with higher cognitive performance—specially based on nonverbal skills—exhibited weaker expression of the FC pattern, mainly loaded by DAN‐SMN (network strength = 0.76) and DAN‐VIS (network strength = 0.73) between‐network pairs. The FC‐sensory rCCA revealed a canonical pair (*r* ^2^ = 0.63, p_FDR_ = 3.4e−12) explaining 62.8% of sensory processing variance, as well as 11.3% of FC variance (Figure 6B). Auditory, tactile and proprioception were the most contributing modalities to the sensory canonical variate. This dimension was mainly correlated with higher FC in ASD children between DAN‐SMN networks (network strength = 1.58). Finally, the most powerful canonical pair (*r* ^2^ = 0.54, p_FDR_ = 5.8e−9) yielded by the FC‐Social rCCA explained 84.7% and 10.7% of SRS and FC variance, respectively (Figure 6C). The social canonical dimension was characterized by high loadings of social motivation and restricted and repetitive behavior subscales. ASD children exhibited higher values in this social dimension that were associated with increased FC prominently between DAN‐FPN (network strength = 1.24) and DAN‐SMN (network strength = 1.17) networks.

## 4 Discussion

In the present study, we used source‐level reconstruction of passive non‐task‐related HD‐EEG to characterize functional network overconnectivity and its association with cognitive, sensory, and social dimensions in young children with ASD. Compared to a group of TD children, cortical network metrics in the alpha band of ASD children revealed local overconnectivity in frontotemporal and lateral occipital regions. A frontotemporal cross‐hemispheric FC pattern that consistently and reliably differentiated between ASD and TD children. Finally, we identified three latent FC–behavior dimensions that capture individual differences in cognitive, social, and sensory features. Although each linked brain–behavior dimension described a distinct FC pattern, overconnectivity between DAN and SMN networks was a recurrent pattern contributing to explain variance in ASD‐related behavioral dimensions. Taken together, these findings delineate discrete patterns of cortical functional overconnectivity that are associated with individual differences in cognitive, social, and sensory ASD‐related manifestations.

Given the acknowledged limitations of univariate methods to establish generalizable brain–behavior dimensions (Rosenberg and Finn 2022), data‐driven multivariate approaches provide a reasonable alternative to explore solid relationships between brain features and behavior (Wang et al. 2021; Buch et al. 2023). We conducted rCCA to build up maximal correlations between linear combinations of both behavioral and FC data to better understand how atypical network overconnectivity underlies individual differences in ASD‐related phenotypic manifestations. The cortical network overconnectivity most strongly correlated to individual variability in the cognitive dimension was between DAN and SMN networks, as well as between DAN and VIS networks. These results are in agreement with previous resting‐state fMRI evidence of a robust association between higher intelligence scores and lower FC in connections between cortical areas comprising DMN and DAN, including the visual and somatosensory regions (Hearne et al. 2016; Dryburgh et al. 2020). FC patterns contributing to variance in the dimension based on social responsiveness involved increased functional interactions mainly between DAN, FPN, and SMN networks. It is important to note that similar patterns of FC‐behavior relationships have already been observed in ASD individuals, with higher SRS scores associated with hyperactivity in several DAN and FPN regions relevant for socio‐emotional processing (Elton et al. 2016). There is compelling evidence indicative of reduced segregation of social brain networks in ASD, manifested as an excessive number of connections with irrelevant regions (Muller and Fishman 2018). Finally, greater auditory, tactile, and proprioceptive processing difficulties in ASD young children were associated with higher FC between DAN and SMN networks. These results are in line with previous work that demonstrates increased FC of primary sensory networks in ASD individuals (Cerliani et al. 2015). Other studies have established robust relationships between sensory and social responsiveness and FC in salience networks (Green et al. 2016; Minnigulova et al. 2025), but we could not replicate this result with our sample. Recurrent overconnectivity between DAN and SMN networks was shared between all identified brain‐behavior dimensions, consistent with previous work showing hyper‐connectivity in both dorsal and ventral attentional networks of young ASD children (Farrant and Uddin 2016).

A prevailing theory of developmental FC changes in ASD posits that young ASD children exhibit local overconnectivity, in contrast to underconnectivity in adults with ASD (Kana et al. 2014; Keown et al. 2013). Here we expand upon previous findings of increased FC in ASD early childhood by characterizing higher‐level network properties using tools derived from the physics of complex networks (Rubinov and Sporns 2010). We report local network overconnectivity in community organization within frontotemporal cross‐hemispheric networks and in lateral‐occipital regions, in young children with ASD relative to their TD counterparts. It is hypothesized that FC within frontal lobe of ASD individuals is excessive, with disorganized and inadequately selective network properties, whereas FC with other lobes is suboptimal, with reduced responsiveness and deficient transfer of information (Courchesne and Pierce 2005; Belmonte et al. 2004). We also observed that frontotemporal networks in young children with ASD had shorter average path lengths (i.e., higher levels of local efficiency). Randomly connected networks tend to have shorter path lengths (Sporns 2011), suggesting the possibility that higher efficiency may simply reflect a less organized or more random distribution of functional edges. This is consistent with a study finding decreased complexity or increased randomness in resting‐state fMRI timeseries of individuals with ASD (Lai et al. 2010). Our node‐wise analysis also revealed increased regional segregation in the lateraloccipital and inferior parietal regions. Similar patterns have been observed with other neuroimaging techniques (Nair et al. 2018; Jao Keehn et al. 2019; Kitzbichler et al. 2015), suggesting region‐specific overconnectivity partially associated with more integrative long‐distance FC. Furthermore, local overconnectivity in occipital and posterior temporal regions of children with ASD has been reported (Lanciano et al. 2025), together with underconnectivity in posterior cingulate and medial prefrontal regions (Maximo et al. 2013).

A key interpretive consideration in case–control studies with ASD children is the role of cognitive ability, closely intertwined with the autistic phenotype. IQ differences between groups are common in developmental autism cohorts and reflect the inherent heterogeneity of the condition. While controlling for IQ is important to isolate diagnosis‐specific effects, it also carries the risk of removing variance that is intrinsically meaningful to the autistic phenotype. Our results speak directly to this tension. When including FSIQ as a covariate, the overall pattern of node‐wise differences remained largely unchanged. A small subset of regional effects, primarily in posterior regions (e.g., pericalcarine cortex and precuneus), no longer reached significance. The attenuation of effects in these regions suggests that part of the variance in posterior connectivity may be shared with general cognitive functioning or perceptual processing, rather than genuine core group differences. Global network topology differences also remained statistically robust after controlling for FSIQ, suggesting that these macroscale organizational properties are not secondary to cognitive level differences between groups. In contrast, the edge‐wise subnetwork identified by NBS was substantially reduced after FSIQ adjustment, indicating that a portion of the local hyperconnectivity observed at the connection level is shared with or amplified by cognitive differences. Taken together, these findings suggest a dissociation between local, edge‐wise connectivity differences that are partially sensitive to IQ, and global network topology alterations that are more intrinsic to the ASD phenotype. The reduction in subnetwork extent after covariate adjustment underscores the risk of overestimating the specificity of connectivity differences when IQ is not accounted for.

We found higher relative power in the alpha band in young children with ASD. At rest, the EEG signal is characterized by oscillations in the alpha frequency band, which have been associated with attention and perceptual processing tasks (Knyazev et al. 2006). Neural activity in the alpha band is of interest since it is thought to denote the level of cortical excitability (Sauseng et al. 2009). The neural oscillatory pattern exhibited by ASD children in our study seems consistent with previous studies (Chan and Leung 2006; Ogawa et al. 1982), and has been associated with cortical deactivation or inactivity (Sauseng et al. 2009; Rihs et al. 2007). Age‐dependent alpha FC patterns have been observed, demonstrating hyperactivation in the ASD group for ages below 6–7 years old and hypoactivation for older ages (Kurkin et al. 2023). However, there is also contrasting evidence suggesting comparable alpha spectral density between children with ASD and TD children (Coben et al. 2008). The potential sources of these discrepancies may include differences in the experimental protocols and methodology. For instance, higher levels of posterior alpha activity have been observed in ASD individuals during eyes‐open resting‐state EEG, seemingly attributable to reduced occipital alpha suppression (Mathewson et al. 2012).

Classically, FC has usually been computed at the scalp level, not allowing interpretation of anatomically interacting brain areas as they are severely corrupted by the volume conduction effects (Brunner et al. 2016). Here, the EEG source connectivity method was used to identify functional networks at the cortical level from HD‐EEG recordings (Hassan et al. 2014). As compared with fMRI studies, a unique advantage of this method is that networks could be directly identified at the cerebral cortex level from scalp EEG recordings, which consist of direct measurement of neuronal activity, in contrast with blood‐oxygen‐level‐dependent (BOLD) signals. This method was first evaluated for its capacity to reveal relevant networks in a picture naming task (Hassan et al. 2014), and was then extended to the tracking of the spatiotemporal dynamics of reconstructed brain networks (Hassan et al. 2015). HD‐EEG offers several practical advantages as a non‐invasive, portable, widely available, and cost‐effective technology with good clinical practicality. Compared with MRI, EEG may have higher relative tolerance for movement, higher temporal resolution, and could be operated with a lower level of expertise at a considerably lower cost (Biasiucci et al. 2019).

Our study has several limitations. First, methodological differences related to eye status (open/closed) must be considered. Local occipital overconnectivity in eyes‐open states has indeed been found in several independent ASD cohorts (Nair et al. 2018; Washington et al. 2014), and data from participants with eyes closed was recently shown to heavily impact regional homogeneity patterns, particularly in the occipital lobe (McAvoy et al. 2008). In our study, EEG recordings were acquired during passive viewing of silent videos to ensure compliance in young children. While this approach is widely used in pediatric neuroimaging (Wilkinson et al. 2024), it may introduce variability related to attention or gaze patterns, particularly in autism. Future studies combining EEG with eye‐tracking will be important to disentangle intrinsic connectivity from stimulus‐driven effects. Movie stimuli do indeed elicit inter‐subject correlations (ISC)—that is well documented in fMRI and EEG studies (Hasson et al. 2008). Yet large‐scale functional organization measured during naturalistic viewing overlaps strongly with resting‐state networks, with differences largely reflecting attention/engagement rather than wholesale reconfiguration (Sanchez‐Alonso et al. 2021). Thus, functional connectivity estimated during movies can still index intrinsic organization relevant to individual differences (Wang et al. 2017; Gal et al. 2022). Also, our study restricts inferences to FC in the alpha band, constraining generalizability across frequency space. A frequent challenge when computing FC at the source level is the “spatial leakage” as the reconstruction of true dipole sources from the scalp signals will be spread over numerous voxels. Here, the choice of the inverse solution/connectivity combination was supported by comparative studies using simulated data from a biophysical/physiological model (Hassan, Merlet, et al. 2017), and real data recorded during a cognitive task (Hassan et al. 2014). Also, an attrition rate of approximately 25% in our EEG dataset for the ASD children represents a non‐trivial limitation. However, it must be emphasized that working with young autistic children is inherently challenging when maintaining attention, tolerating EEG caps, and minimizing motion artifacts. Such dropout rate is therefore not unexpected and is consistent with rates pre‐identified and reported in prior studies (Webb et al. 2019). Importantly, effect sizes observed at the group level were generally small to moderate, consistent with the heterogeneity of autism. The present study was not designed to achieve diagnostic classification, but rather to identify dimensional relationships between brain connectivity and behavioral domains. Future work may explore whether multivariate approaches can improve individual‐level prediction. Finally, the sample size of our ASD group is relatively limited and we did not replicate our findings in an independent dataset. Further growth of public datasets in this particular age range has been challenging, but recent efforts will surely provide opportunity for cross‐validation and stronger statistical power.

## 5 Conclusion

In summary, here we used an innovative HD‐EEG source reconstruction model to show that cortical network metrics in young children with ASD are characterized by specific overconnectivity‐guided dimensions contributing to distinctive ASD‐related behavioral features. Collectively, the stratification of well‐defined neural signatures that give rise to the clinical heterogeneity of ASD has the potential to provide more accurate prognosis and help to select the optimal strategy for therapeutic intervention.

## Funding

This project was funded by the F. Hoffmann‐La Roche.

## Conflicts of Interest

M.H. is a full‐time employee in MINDIG company. The remaining authors declares no conflicts of interest.

## Supporting information


**Figure S1:** Functional network parcellation of the brain. The cortical areas were parcellated with 68 seed regions, and these seed regions were assigned to 8 macroscale cortical networks: visual network (VIS), auditory network (AUD), sensorimotor network (SMN), fronto‐parietal network (FPN), salience network (SAN), dorsal attentional network (DAN), default mode network (DMN), and other networks (OTHER). The corresponding information of each seed region is shown in Table S1.
**Figure S2:** Heatmaps used to select regularization parameters for the FC‐Global cognition (A), FC‐Social cognition (B) and FC‐Sensory processing (C) rCCA. We used the Cross‐Validation Ridge method, where the highest cross‐validated score is desired, in a constrained range between 0.001 and 0.2 (length = 10) to yield the highest canonical correlation of the first variate.
**Table S1:** Affiliation of the 68 cortical regions –derived from the Desikan‐Killiany atlas–, to the eight large‐scale resting‐state cortical networks.
**Table S2:** SRS‐2 and SPM subscales T‐scores of the study cohort of TD and ASD children expressed as Mean (SD).

## Data Availability

Consents obtained from our participants prevent sharing individual data without a data use agreement in place. Please contact the corresponding author with data requests. The code used for the EEG processing and analyses is publicly available at https://github.com/stsa‐research.
