# Supplementary material for: Cortical Network Overconnectivity Relates to Sensory, Cognitive, and Social Dimensions in Young Children With Autism Spectrum Disorder
Source: Autism Res. 2026 Jul 17;19(9):e70315. doi: 10.1002/aur.70315 (PMC13599687; doi:10.1002/aur.70315)
Supplement: Supplementary file 1 [file AUR-19-0-s001.docx]

**Supplementary Materials**

**Cortical network overconnectivity relates to sensory, cognitive, and social dimensions in young children with autism spectrum disorder**

Borja Rodríguez-Herreros^1^†, Ahmad Mheich^1^†, Joana Maria Almeida Osório^1^, Sonia Richetin^1^, Vincent Junod^2^, Lorène Arnold^1^, Victoria Aeschbach^1^, Lilia Nora Adamou^1^, Katherina Gschwend^1^, Laura Mendes^1^, David Romascano^1^, Paola Yu^1^, Marine Jequier Gygax^1^, Anne M. Maillard^1^, Micah M. Murray^3,4^, Mahmoud Hassan^5,6^* and Nadia Chabane^1^*

**†These authors contributed equally to this work.**

***These authors contributed equally to this work.**

*^1^Service des Troubles du Spectre de l’Autisme et apparentés, Département de psychiatrie, Lausanne University Hospital (CHUV), Lausanne, Switzerland*

^2^*Réseau fribourgeois de santé mentale (RFSM), Département de psychologie, 1762 Givisiez, Switzerland*

^3^*Department of Radiology, Lausanne University Hospital and University of Lausanne, Lausanne, Switzerland.*

^4^*The Sense Innovation and Research Center, Lausanne and Sion, Lausanne, Switzerland.*

^5^*MINDIG, Rennes, F-35000, France*

^6^*School of Science and Engineering, Reykjavik University, Reykjavik, Iceland*

Correspondence to: **Borja Rodríguez-Herreros, PhD**

Service des Troubles du Spectre de l'Autisme & apparentés

Centre Cantonal de l'Autisme

Les Allières, Al/05/508

Av. de Beaumont 23, CH-1011 Lausanne
Tél. +4121 314 36 91/ Fax +41 21 314 48 87

**ORCID ID** : 0000-0001-9392-7996

[borja.rodriguez-herreros@chuv.ch](mailto:borja.rodriguez-herreros@chuv.ch)

The supplementary material has been provided by the authors to give readers additional information about their work.

1. **PHENOTYPIC EVALUATION**

**Overall cognitive functioning.** We used two different tests to measure overall cognitive abilities (intelligence quotient, IQ) in children from 2 years 6 months to 10 years of age: The Wechsler Preschool and Primary Scale of Intelligence (WPPSI-IV)^1^ and the Wechsler Intelligence Scale for Children, 5th edition (WISC-V)^2^. Both test batteries included verbal and nonverbal subscales. We used full scale (FSIQ), verbal (VIQ) and nonverbal IQ (NVIQ) standardized to a mean of 100 and a SD of 15, as the outcome measures of cognitive level. For younger children and those not being able to comply with the Wechsler scales, we used the Mullen Scales of Early Learning (MSEL)^3^. We used MSEL Visual Reception subscale as the outcome measure for global nonverbal abilities. In the ASD group, 11 individuals were evaluated with MSEL, 8 with WISC-V and 19 with WPPSI-IV. Finally, 9 TD were assessed with the MSEL, 24 with WISC-V and 33 with WPPSI-IV.

**Social Responsiveness Scale.** In addition to a Total score reflecting a unitary construct of the severity of social deficits, SRS-2 also provides five subscale scores that reportedly constitute treatment subscales including Social Awareness, Social Cognition, Social Communication, Social Motivation, and Mannerisms. We used normally distributed, standardized T-scores, interpreted as: ≤ 59T, within normal limits; 60–65 T, mild; 66–75 T, moderate; ≥ 76T severe^4^.

**Sensory processing questionnaire**. The age range for SPM-P and SPM was 2–5 and 5–12 years old, respectively. Both SPM-P and SPM include 75 items divided into eight subscales: Social Participation (SOC), Planning and Ideas (PLA), Vision (VIS), Hearing (HEA), Touch (TOU), Taste and Smell (TAS), Body Awareness (BOD) and Balance and Motion (BAL). The last two subscales refer to internal sensory modalities —proprioception and vestibular system—, respectively. A raw total Sensory Score (TOT) was calculated based on the raw score of six sensory system subscales (VIS, HEA, TOU, TAS, BOD and BAL). SOC and PLA refer to higher integrative functions (social functioning and praxis, respectively) and hence did not contribute to the composite score. Standardized T-scores for each subscale except for TAS were used as outcome measures. SPM was used for a sample of 16 ASD and 33 TD children. Twenty-two ASD individuals and 33 TD were assessed with SPM-P version.

2. **EEG PRE-PROCESSING WITH AUTOMAGIC TOOLBOX**

The EEG system uses the vertex (Cz) as physical reference. Prior to place the cap on the child’s head, the net was soaked in an electrolyte solution to facilitate electrical contact between the scalp and the electrodes. Individual sensors were adjusted until impedances were kept below 50 kΩ. All channel signals were amplified using NetAmps 200 amplifier and digitized with a 12-bit A/D converter. A channel was labelled as error-prone based on three criteria: i) when the correlation between the recorded data and an estimate of neighboring electrodes was r<0.85; ii) if it has more line noise relative to its signal than all other electrodes (4SD); and iii) if it exhibits a flatline for longer than 5 sec. Next, data were filtered using a 0.1Hz high-pass filter with a cutoff frequency of -6dB, and a ZapLine algorithm was applied to line noise artifacts^5^. To further remove noise contribution to the signal, electro-oculography (EOG) regression effectively mitigated artifacts caused by eye movements and separated them from the HD-EEG signal using the subtraction method that relies on linear transformation of the HD-EEG activity^6^, resulting in a reduction of the final number of channels to 118. After the artifact correction, residual bad channels were excluded if their SD exceeded a threshold of 20μV and/or if they showed a change of amplitude of ±80 μV at any temporal point. Subsequently, Automagic pipeline automatically assessed the quality of the resulting HD-EEG signals based on four criteria: i) if the rate of overall high-amplitude (OHA, >30 μV) data points in the signal was larger than 0.2; ii) if more than 20% of timepoints showed a variance larger than 15μV across channels; iii) if more than 30% of channels showed high variance (>15μV); and iv) if the rate of error-prone electrodes was higher than 0.15. In the next step, error-prone electrodes were removed and later interpolated using an EEGLab spherical spline interpolation.

3. **SOURCE RECONSTRUCTION**

To obtain estimates of brain activity at the cortical level, we applied the first step of the EEG source connectivity method^7^. We reconstructed the dynamics of cortical brain sources from the artifact-free HD-EEG data segments by solving the inverse problem. An atlas-based approach was used to project HD-EEG sensor signals onto an anatomical framework consisting of 68 cortical regions identified by means of the Desikan-Killiany cortical atlas^8^ using Freesurfer^9^. The number of cortical regions aligns optimally with the spatial resolution of EEG source estimates^10^, facilitating robust registration in pediatric brains. We used age-appropriate pediatric MRI templates obtained from the McConnell Brain Imaging Centre database^11^. To account for developmental differences in head and brain anatomy, templates were selected based on participant age: children older than 4.5 years were modeled using the pediatric atlas spanning 4.5–18.5 years, whereas children younger than 4.5 years were modeled using the infant atlas (0–4.5 years). These templates are derived from longitudinal structural MRI data and provide realistic representations of age-specific brain volume, head geometry, and tissue morphology. The use of age-stratified templates improves the accuracy of the forward source localization model in developmental populations while maintaining a standardized framework across participants. Using the OpenMEEG toolbox^12^, the procedure included the co-registration of the pre-processed HD-EEG signal with the MRI template through identification of the right/left pre-auricular points and nasion anatomical landmarks. The lead field matrix was then computed for a cortical mesh with 15,000 vertices using Brainstorm^13^. The weighted minimum norm estimate (wMNE)^14^ was used to calculate the temporal dynamics of the 68 cortical regions of interest (ROIs).

4. **NETWORK METRICS**

Weighted undirected networks were constructed in which the connection strength between each pair of nodes was defined as their connectivity value.

Node-wise: The CC of a network can then be calculated as:

$$CC^{w}=\frac{1}{n}\sum_{i\in N} \frac{2t_{i}^{w}}{k_{i}^{w}\left( k_{i}^{w}-1 \right)}$$

Where $t_{i}^{w}$is the number of triangular connections around the node i:

$$t_{i}^{w}=\frac{1}{2}\sum_{j,h\in N} \left( w_{ij}w_{ih}w_{jh} \right)^{\frac{1}{3}}$$

The more the neighborhood of the given node is densely interconnected, the higher is its local CC.

BC is calculated as:

$$BC=\frac{1}{\left( n-1 \right)\left( n-2 \right)}\sum_{h,j\in Nh\neq j,h\neq i,i\neq j} \frac{\varphi_{hj}^{\left( i \right)}}{\varphi_{hj}}$$

Where $\varphi_{hj}$ is the number of shortest paths between node $h$ and node $j$, and $\varphi_{hj}^{\left( i \right)}$ is the number of shortest paths between $h$ and $j$ that pass-through node $i$. BC is the fraction of all shortest paths in the network that contain a given node. Nodes with high BC values participate in a large number of shortest paths. To compute BC, we first calculated the shortest path length (SPL) between nodes, that is, the minimum number of links to be traversed in the optimal path from one node to the other.

LE is calculated as:

$$LE=\frac{1}{n\left( n-1 \right)}\sum_{j,k\in N} \frac{1}{L_{j,k}}$$

Where $L_{j,k}$ is the average distance (number of steps) between nodes $j$ and $k$ in the network.

Global topology: SWP is defined as:

$${SWP}^{w}=\frac{\frac{CC}{CC_{rand}}}{\frac{SPL}{{SPL}_{rand}}}$$

Where CC and $CC_{rand}$ are the clustering coefficients, and $SPL$ and ${SPL}_{rand}$ are the characteristic path lengths of the tested and the random network, respectively. SWP evaluates the level of small-world structure between any two nodes, that is, higher local clustering than a random network, yet short average path length.

GE measures the efficiency of information transfer among all pairs of nodes in the graph, and thus reflects the efficiency of interaction across the whole graph^15,16^.

EWCI is defined as the sum of the weights of the resultant subnetwork:

$$EWCI=\left( \sum_{e}^{M} w_{e} \right)\times100$$

where $w_{e}$ represents the weight of the edge $e$ in the subnetwork and $M$ is the number of edges.

5. **REGULARIZED CANONICAL CORRELATION ANALYSIS (RCCA)**

Five participants were excluded from the rCCA due to incomplete clinical data, leaving the final sample in 99 children (64 TD vs 35 ASD). The input to the rCCA were the 32 network-level dimensions from the list of ranked FC features and the following phenotypic measures: Verbal, nonverbal and full-scale IQ for the cognitive domain; the five SRS subscales T-scores (Awareness, Cognition, Communication, Motivation, Mannerisms) plus the total SRS T-score for the social domain; and the seven SPM subscales T-scores (Social, Visual, Hearing, Touch, Body, Balance, Planning) plus the total SPM T-score for sensory processing domain. We pooled all participants to estimate transdiagnostic dimensions of covariation between behavior and FC. Pooling increases the sample-to-feature ratio, stabilizes the canonical axes, and yields a single latent space in which individual differences can be compared. To prevent group mean differences in both the FC and the phenotypic data from driving the solution, features were z-scored within group, age/sex were residualized, and a group indicator was included as a covariate. The L2-penalty was settled for both variable sets (FC features and clinical measures) on a hyperparameter grid of λ_FC_ = [1,2,3,4,5] and λ_cog_ = [0.002,0.13], λ_srs_ = [0.002,0.02] and λ_sens_ = [0.002,0.2], respectively.

Before running the rCCA, we used the Spearman correlation between FC and the standard clinical measures in 50 subsampled replicates. We ranked FC features by how frequently they were selected as statistically significant, and used this rank list as input into rCCA to estimate sets of linear combinations of FC features that best relate to linear combinations of clinical features. We then generated a coarse grid of possible values for λ1 and λ2 and determine the optimal regularization parameters. Model performance was evaluated using a cross-validation approach, based on robust null permutation approach in 1,000 training set/test set replicates, with test data (15%) held out from feature selection and rCCA calculation.

Loadings of the canonical variates represent the weights assigned to each of the original variables to determine their contribution to a given latent dimension. The canonical loadings are crucial for the interpretation step as higher loading values indicate greater contribution of the given variable to the canonical dimension. We reported canonical dimensions that were statistically significant at false discovery rate correction (FDR, q < 0.05) and which explain more than 5% of the covariance for further analyses^17^.

**Supplementary figures**

**Supplementary Figure S1.** Functional network parcellation of the brain. The cortical areas were parcellated with 68 seed regions, and these seed regions were assigned to 8 macroscale cortical networks: visual network (VIS), auditory network (AUD), sensorimotor network (SMN), fronto-parietal network (FPN), salience network (SAN), dorsal attentional network (DAN), default mode network (DMN) and other networks (OTHER). The corresponding information of each seed region is shown in Table S1.

**Supplementary Figure S2.** Heatmaps used to select regularization parameters for the FC-Global cognition (**A**), FC-Social cognition (**B**) and FC-Sensory processing (**C**) rCCA. We used the Cross-Validation Ridge method, where the highest cross-validated score is desired, in a constrained range between 0.001 and 0.2 (length = 10) to yield the highest canonical correlation of the first variate.

**Supplementary tables**

**Table S1.** Affiliation of the 68 cortical regions –derived from the Desikan-Killiany atlas–, to the eight large-scale resting-state cortical networks

| **Network of Interest** | **Brain regions** |
| --- | --- |
| **Dorsal Attention Network (DAN)** | Caudalanteriorcingulate (L)  Inferiortemporal (L/R)  Middletemporal (L/R) |
| **Auditory Network (AUD)** | Superiortemporal (L/R) |
| **Visual Network (VIS)** | Cuneus (L/R)  Fusiform (L/R)  Lateraloccipital (L/R)  Lingual (L/R) |
| **Frontoparietal Network (FPN)** | Caudalmiddlefrontal (L/R)  Parsopercularis (L/R)  Parsorbitalis (L/R)  Parstriangularis (L/R)  Rostralmiddlefrontal (L/R)  Superiorfrontal (L/R)  Superiorparietal (L/R)  Frontalpole (L/R) |
| **Default Mode Network (DMN)** | Inferiorparietal (L/R)  Isthmuscingulate (L/R)  Lateralorbitofrontal (L/R)  Medialorbitofrontal (L/R)  Parahippocampal (L/R)  Posteriorcingulate (L/R)  Rostralanteriorcingulate (L/R)  Precuneus (L/R)  Supramarginal (L/R) |
| **Somatomotor Network (SMN)** | Paracentral (L/R)  Postcentral (L/R)  Precentral (L/R) |
| **Salience Network (SAN)** | Insula (L/R)  Caudalanteriorcingulate (R) |
| **Other** | Bankssts (L/R)  Entorhinal (L/R)  Pericalcarine (L/R)  Temporalpole (L/R)  Transversetemporal (L/R) |

**Table S2.** SRS-2 and SPM subscales T-scores of the study cohort of TD and ASD children expressed as Mean (SD).

|  |  | **TD (N=66)** | **ASD (N=38)** |
| --- | --- | --- | --- |
| **Social cognition (SRS-2)** | **Awareness** | 45.8 (7.8) | 64.3 (11) |
|  | **Cognition** | 45.2 (5.5) | 70.3 (9.1) |
|  | **Communication** | 45.6 (5.3) | 69.2 (10.4) |
|  | **Motivation** | 46.3 (6) | 64.7 (10.7) |
|  | **Mannerisms** | 45.1 (4.5) | 71.1 (12) |
| **Sensory processing (SPM)** | **Visual** | 50.6 (7.7) | 65.7 (8.1) |
|  | **Auditory** | 52.7 (8.7) | 63.3 (8.7) |
|  | **Tactile** | 51.9 (8.2) | 63.4 (10.7) |
|  | **Proprioception** | 51.7 (7.7) | 61.8 (8.5) |
|  | **Vestibular** | 49.1 (6.9) | 61.8 (9.1) |
|  | **Praxis** | 47.5 (6.8) | 65.4 (7.4) |
|  | **Social participation** | 49.8 (7.6) | 67.1 (7.8) |

ASD: Autism Spectrum Disorder, TD: Typically Developing, SRS-2: Social Responsiveness Scale-2, SPM: Sensory Processing Measure.

**p < 0.05; **p < 0.01; ***p < 0.001*

**References**

1	Wechsler, D. *WPPSI-IV: Échelle d'intelligence de Wechsler pour la période préscolaire et primaire*. (Pearson, 2013).

2	Wechsler, D. (ECPA, Les Editions du Centre de psychologie appliquée Paris, 2014).

3	EM, M. (Mn, 1995).

4	Constantino, J. N. & Gruber, C. P. Social responsiveness scale: SRS-2. *Western Psychological Services* (2012).

5	de Cheveigne, A. ZapLine: A simple and effective method to remove power line artifacts. *Neuroimage* **207**, 116356 (2020). <https://doi.org/10.1016/j.neuroimage.2019.116356>

6	Parra, L. C., Spence, C. D., Gerson, A. D. & Sajda, P. Recipes for the linear analysis of EEG. *Neuroimage* **28**, 326-341 (2005). <https://doi.org/10.1016/j.neuroimage.2005.05.032>

7	Hassan, M. & Wendling, F. Electroencephalography source connectivity: aiming for high resolution of brain networks in time and space. *IEEE Signal Processing Magazine* **35**, 81-96 (2018).

8	Desikan, R. S. *et al.* An automated labeling system for subdividing the human cerebral cortex on MRI scans into gyral based regions of interest. *Neuroimage* **31**, 968-980 (2006). <https://doi.org/10.1016/j.neuroimage.2006.01.021>

9	Fischl, B. FreeSurfer. *Neuroimage* **62**, 774-781 (2012). <https://doi.org/10.1016/j.neuroimage.2012.01.021>

10	Farahibozorg, S. R., Henson, R. N. & Hauk, O. Adaptive cortical parcellations for source reconstructed EEG/MEG connectomes. *Neuroimage* **169**, 23-45 (2018). <https://doi.org/10.1016/j.neuroimage.2017.09.009>

11	Fonov, V. *et al.* Unbiased average age-appropriate atlases for pediatric studies. *Neuroimage* **54**, 313-327 (2011).

12	Gramfort, A., Papadopoulo, T., Olivi, E. & Clerc, M. OpenMEEG: opensource software for quasistatic bioelectromagnetics. *Biomed Eng Online* **9**, 45 (2010). <https://doi.org/10.1186/1475-925X-9-45>

13	Tadel, F., Baillet, S., Mosher, J. C., Pantazis, D. & Leahy, R. M. Brainstorm: a user-friendly application for MEG/EEG analysis. *Comput Intell Neurosci* **2011**, 879716 (2011). <https://doi.org/10.1155/2011/879716>

14	Hamalainen, M. S. & Ilmoniemi, R. J. Interpreting magnetic fields of the brain: minimum norm estimates. *Med Biol Eng Comput* **32**, 35-42 (1994). <https://doi.org/10.1007/BF02512476>

15	de Pasquale, F., Della Penna, S., Sporns, O., Romani, G. L. & Corbetta, M. A Dynamic Core Network and Global Efficiency in the Resting Human Brain. *Cereb Cortex* **26**, 4015-4033 (2016). <https://doi.org/10.1093/cercor/bhv185>

16	Rubinov, M. & Sporns, O. Complex network measures of brain connectivity: uses and interpretations. *Neuroimage* **52**, 1059-1069 (2010). <https://doi.org/10.1016/j.neuroimage.2009.10.003>

17	Modabbernia, A., Janiri, D., Doucet, G. E., Reichenberg, A. & Frangou, S. Multivariate Patterns of Brain-Behavior-Environment Associations in the Adolescent Brain and Cognitive Development Study. *Biol Psychiatry* **89**, 510-520 (2021). <https://doi.org/10.1016/j.biopsych.2020.08.014>
